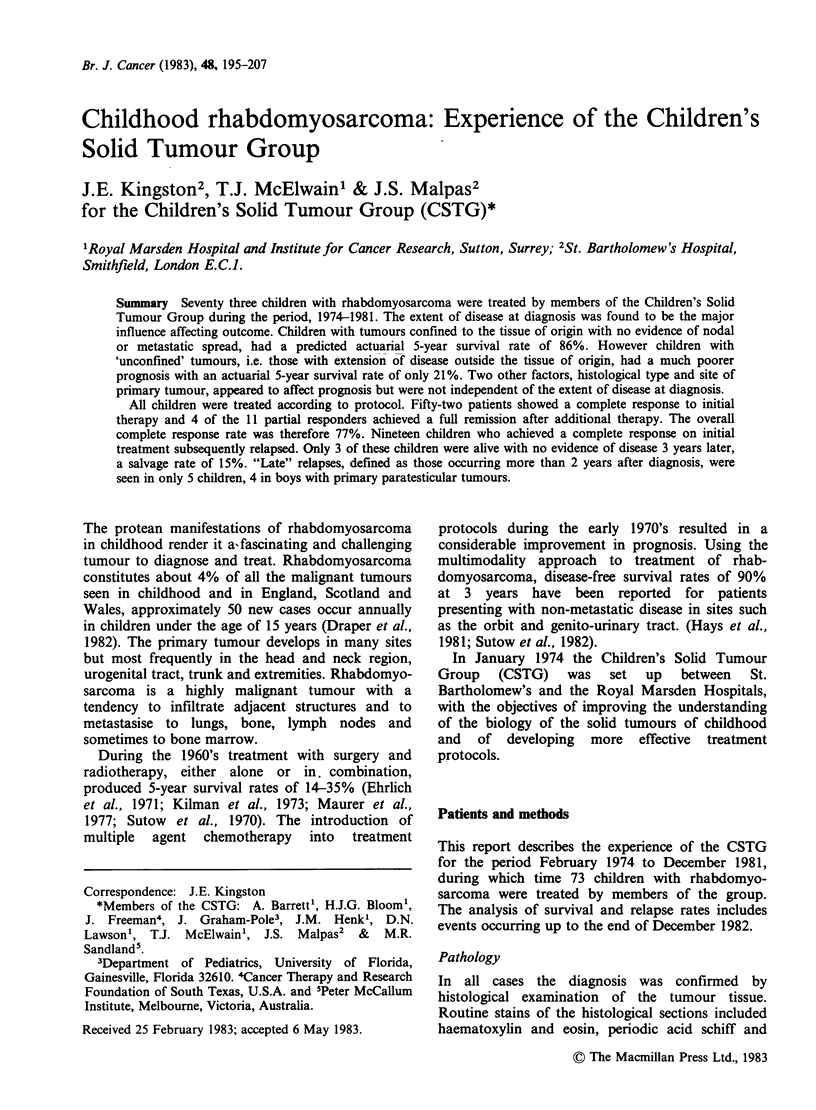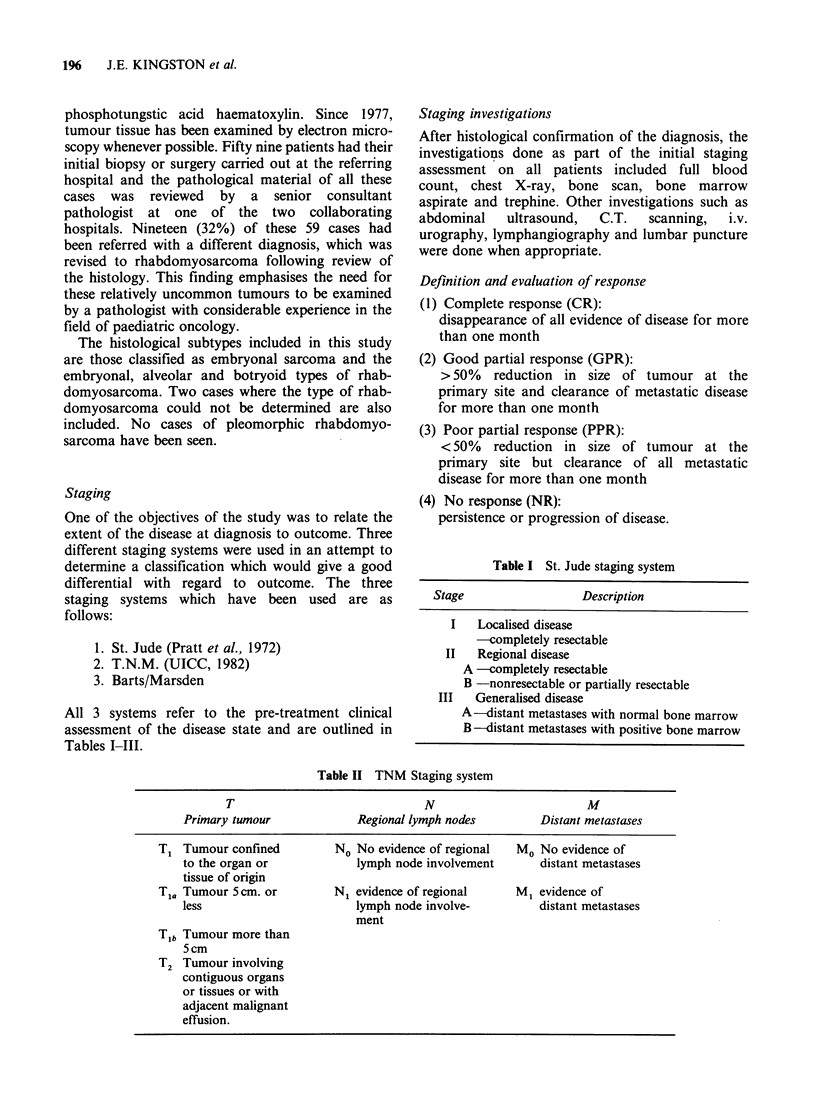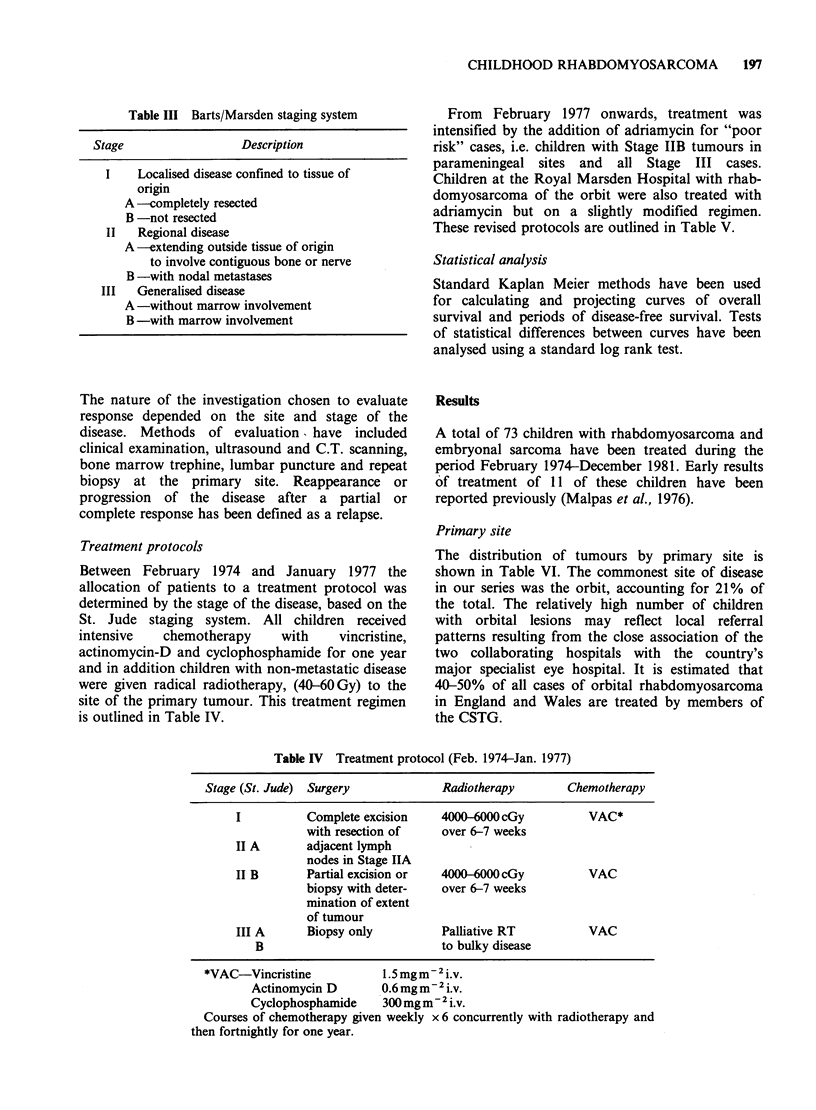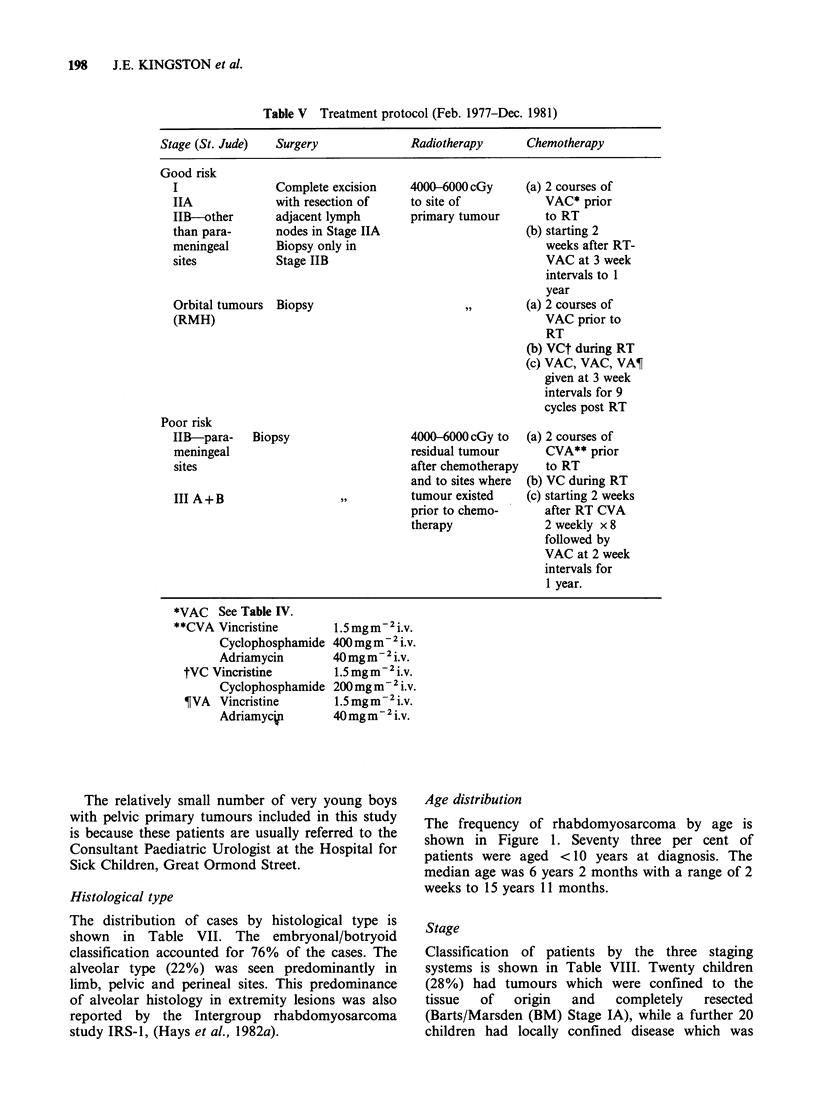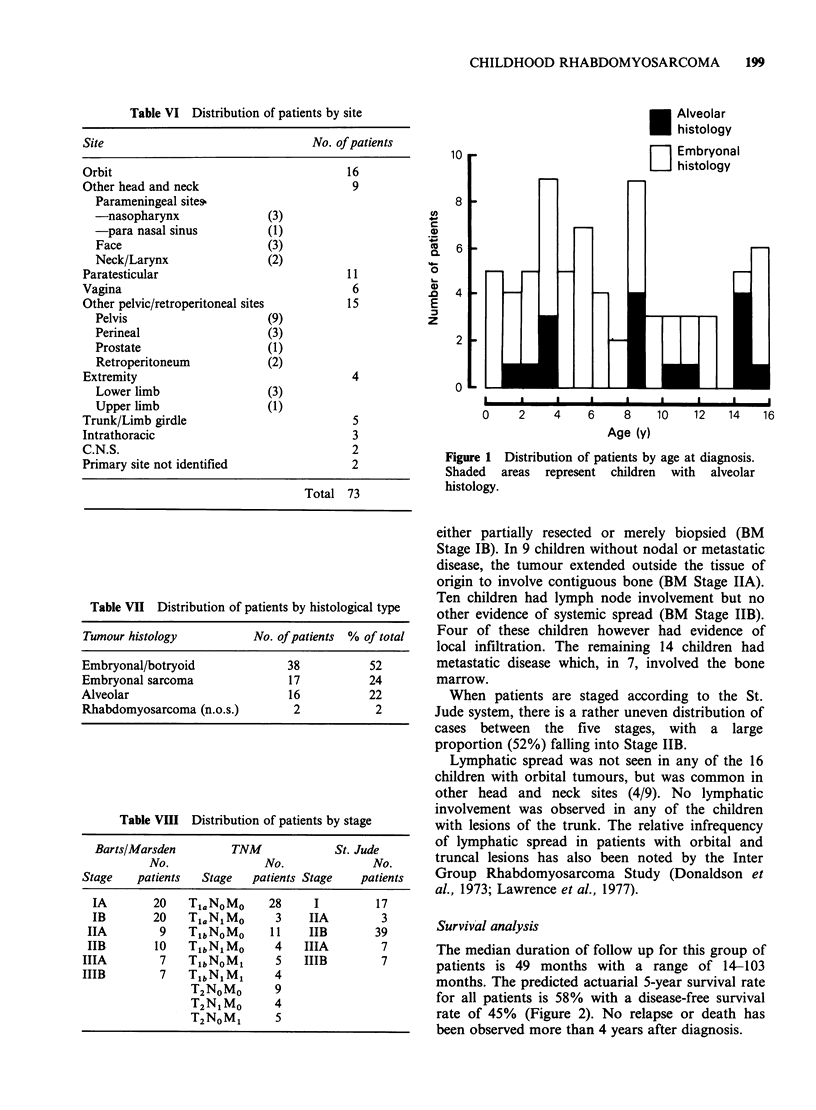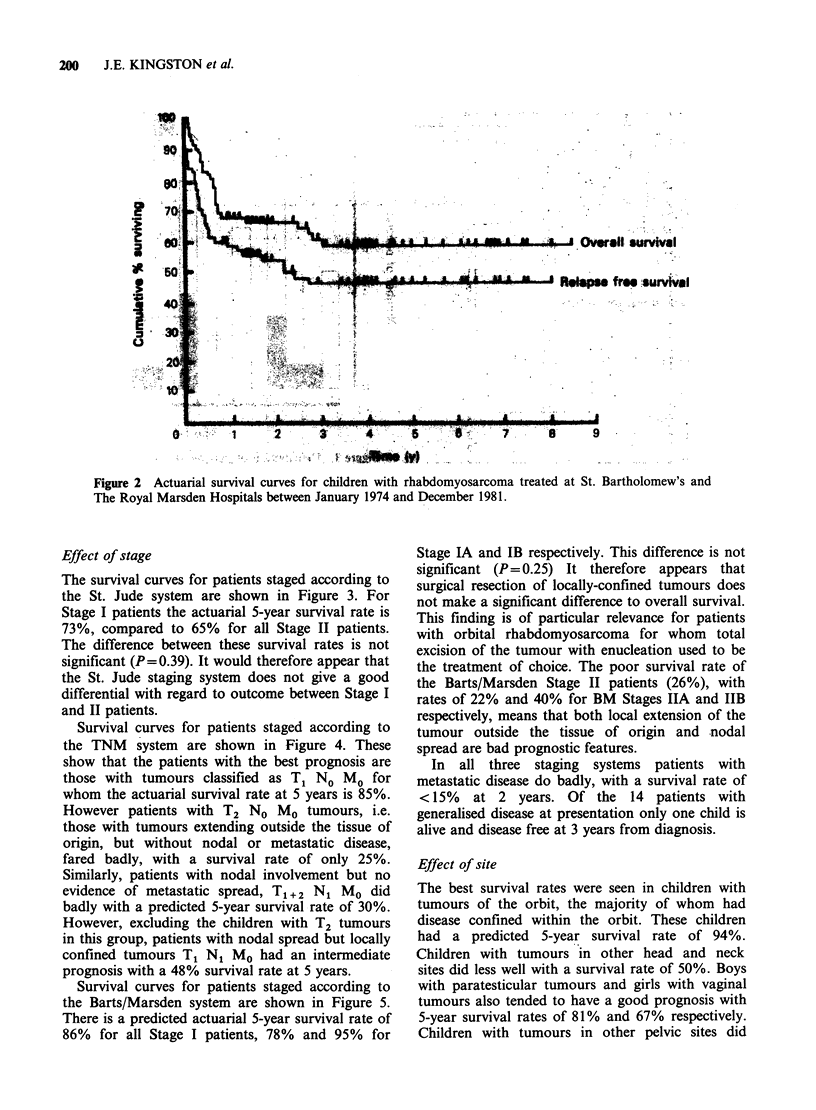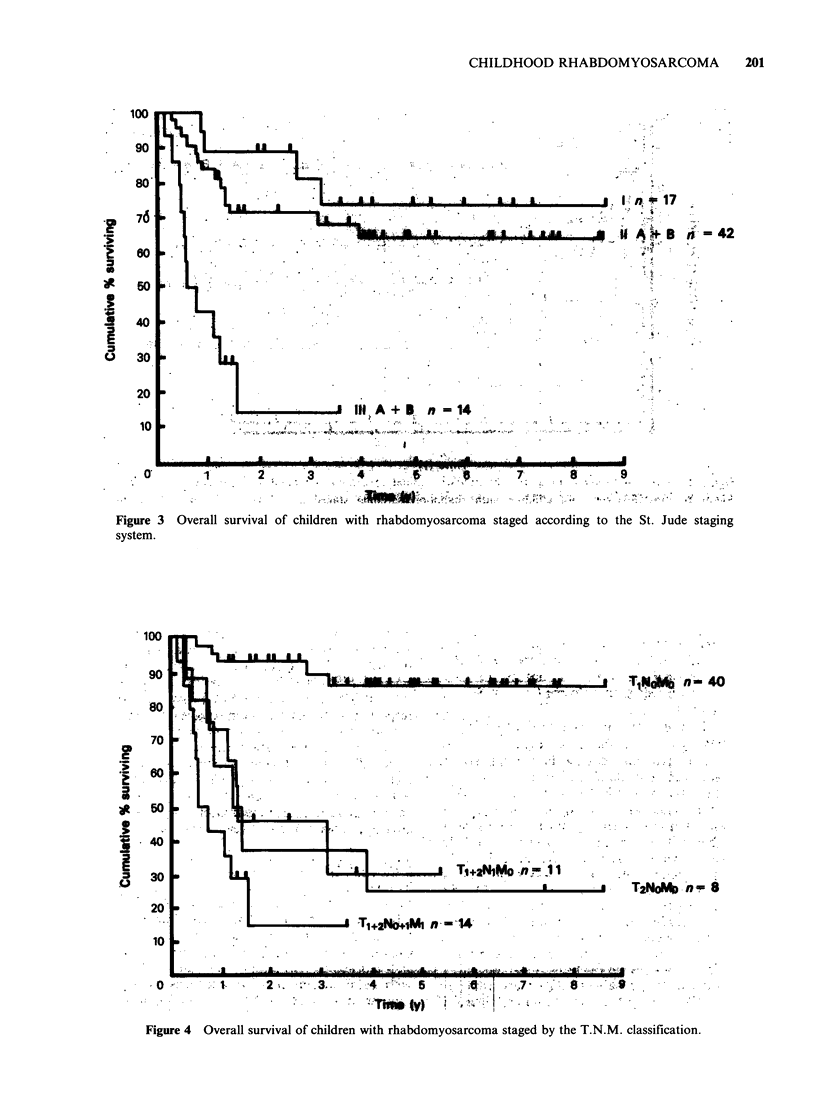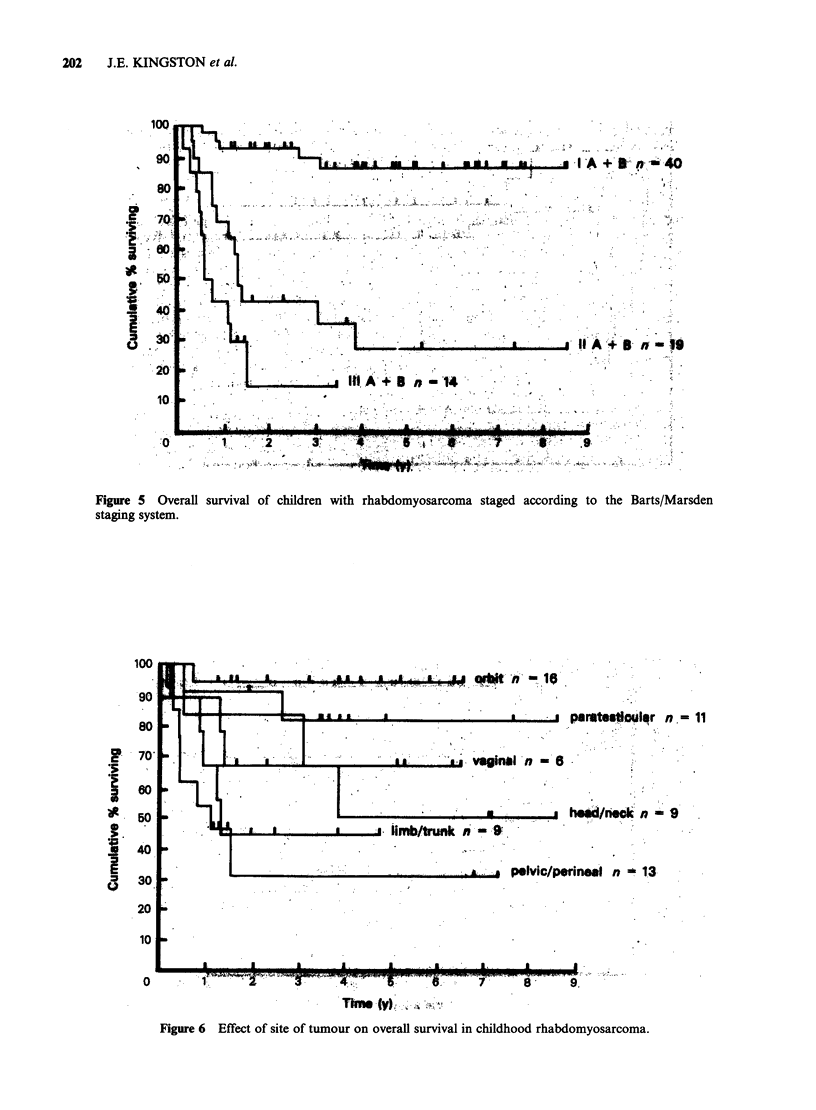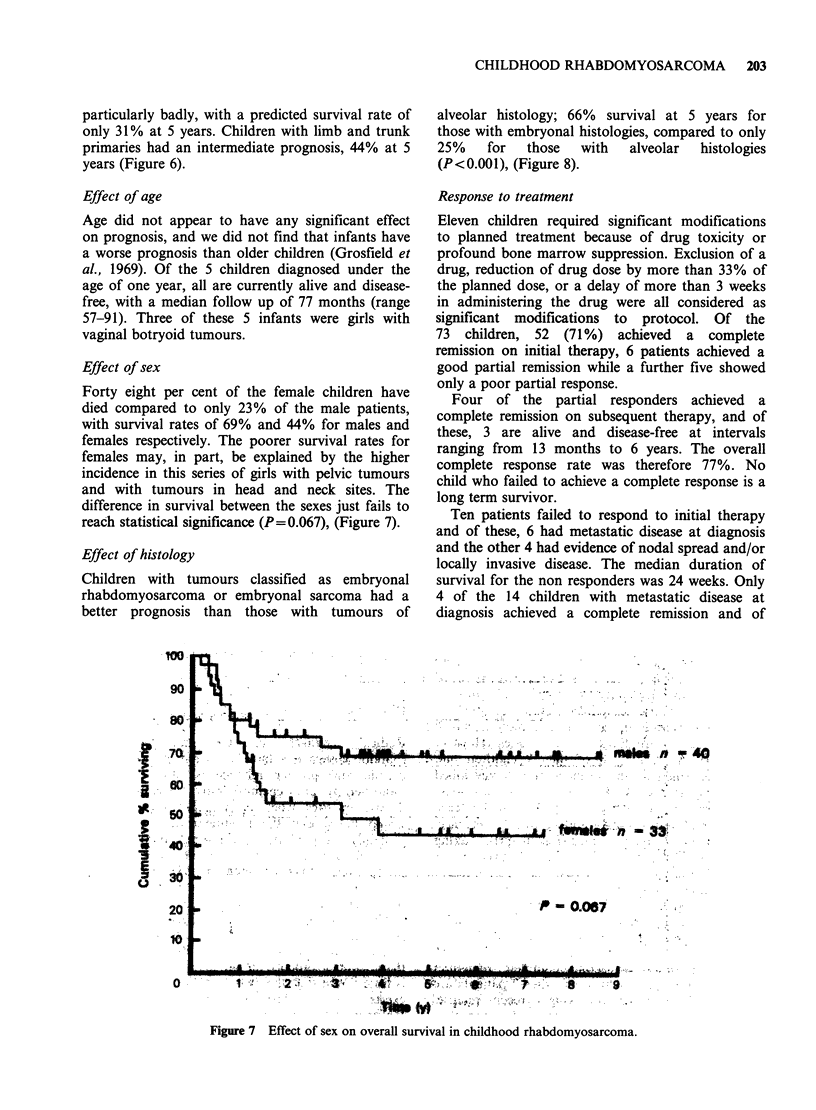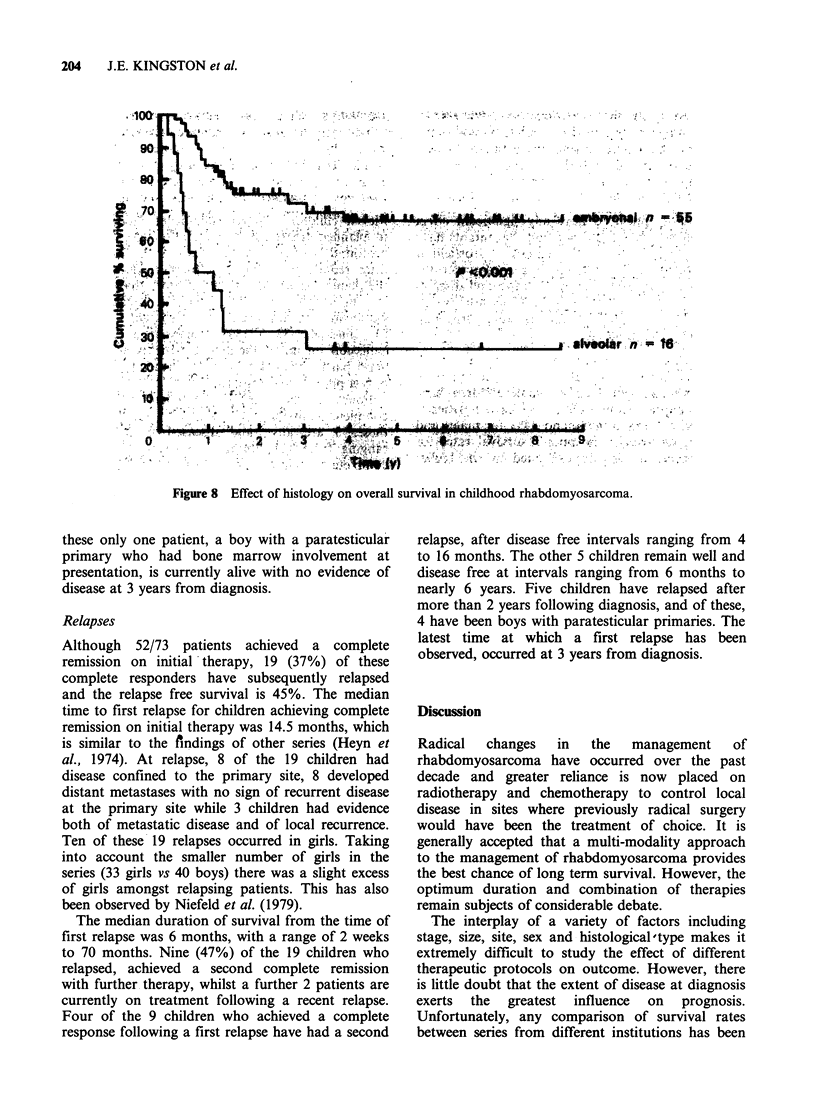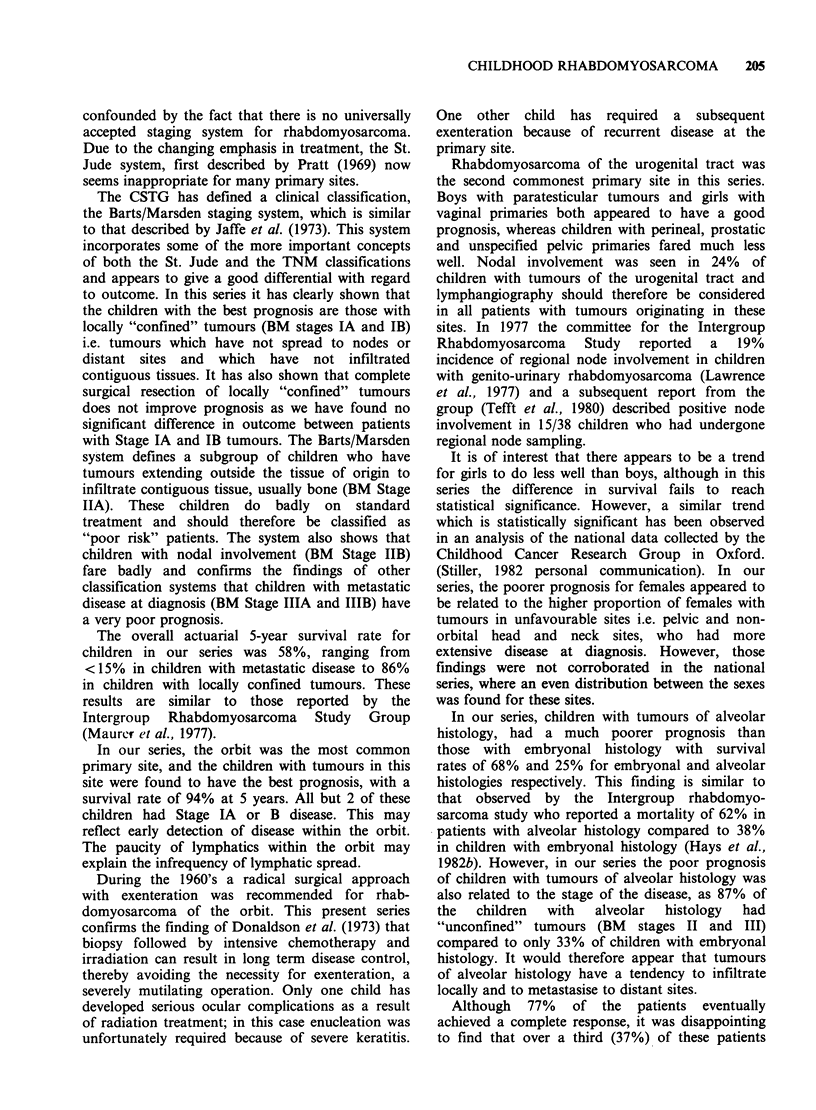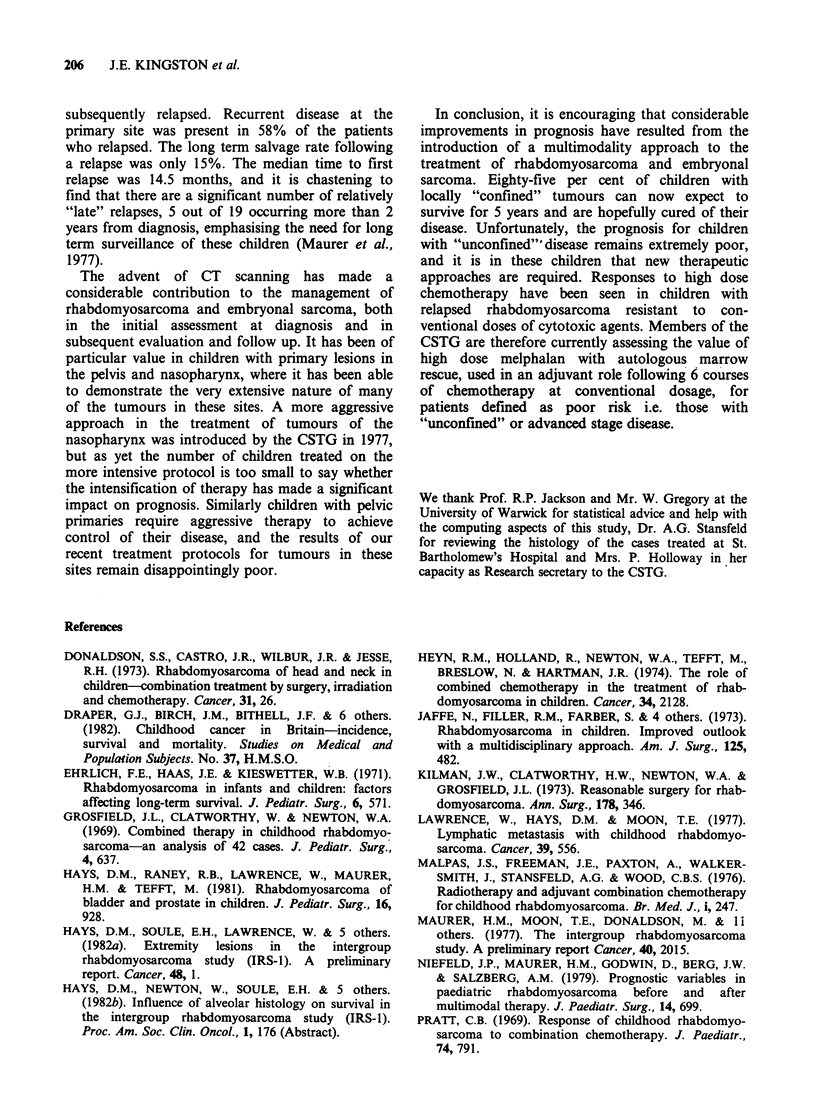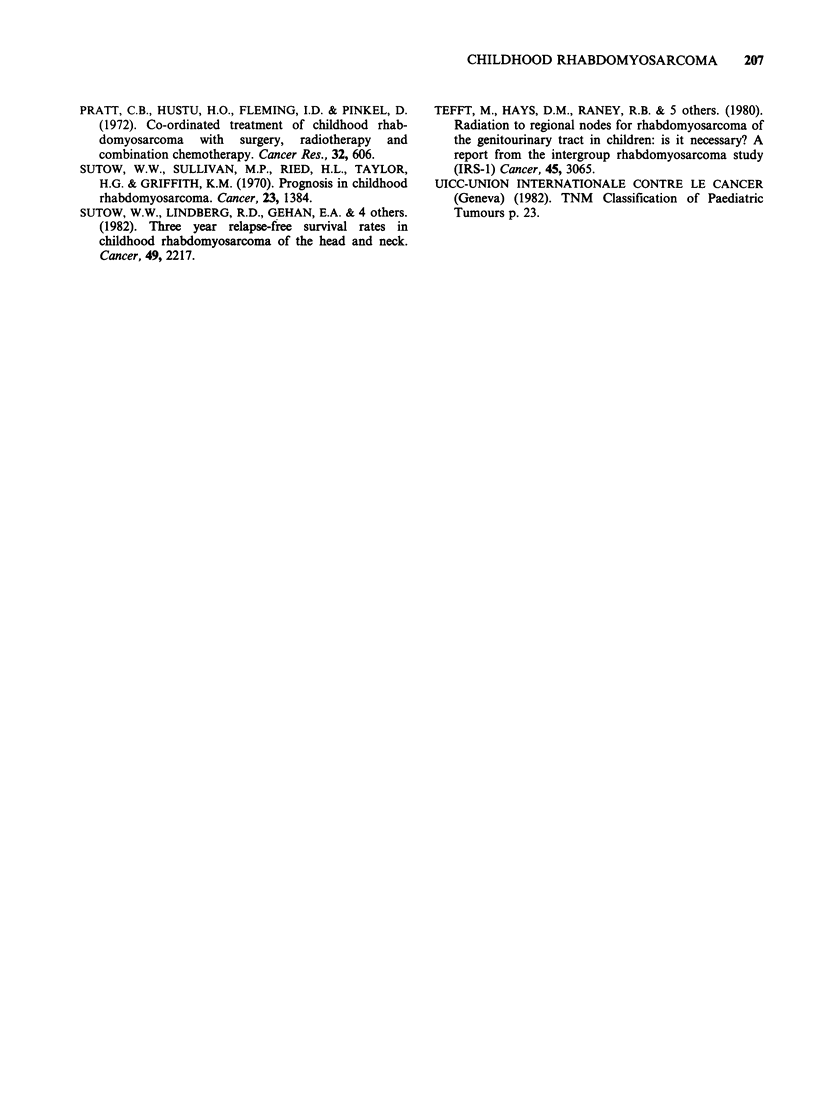# Childhood rhabdomyosarcoma: experience of the Children's Solid Tumour Group.

**DOI:** 10.1038/bjc.1983.175

**Published:** 1983-08

**Authors:** J. E. Kingston, T. J. McElwain, J. S. Malpas

## Abstract

Seventy three children with rhabdomyosarcoma were treated by members of the Children's Solid Tumour Group during the period, 1974-1981. The extent of disease at diagnosis was found to be the major influence affecting outcome. Children with tumours confined to the tissue of origin with no evidence of nodal or metastatic spread, had a predicted actuarial 5-year survival rate of 86%. However children with 'unconfined' tumours, i.e. those with extension of disease outside the tissue of origin, had a much poorer prognosis with an actuarial 5-year survival rate of only 21%. Two other factors, histological type and site of primary tumour, appeared to affect prognosis but were not independent of the extent of disease at diagnosis. All children were treated according to protocol. Fifty-two patients showed a complete response to initial therapy and 4 of the 11 partial responders achieved a full remission after additional therapy. The overall complete response rate was therefore 77%. Nineteen children who achieved a complete response on initial treatment subsequently relapsed. Only 3 of these children were alive with no evidence of disease 3 years later, a salvage rate of 15%. "Late" relapses, defined as those occurring more than 2 years after diagnosis, were seen in only 5 children, 4 in boys with primary paratesticular tumours.


*Br. J. Cancer* (1983), **48**, 195–207

### Childhood rhabdomyosarcoma: Experience of the Children's Solid Tumour Group

J.E. Kingston[2], T.J. McElwain[1] & J.S. Malpas[2]
for the Children's Solid Tumour Group (CSTG)*

[1]*Royal Marsden Hospital and Institute for Cancer Research, Sutton, Surrey;* [2]*St. Bartholomew's Hospital, Smithfield, London E.C.1.*

**Summary** Seventy three children with rhabdomyosarcoma were treated by members of the Children's Solid Tumour Group during the period, 1974–1981. The extent of disease at diagnosis was found to be the major influence affecting outcome. Children with tumours confined to the tissue of origin with no evidence of nodal or metastatic spread, had a predicted actuarial 5-year survival rate of 86%. However children with 'unconfined' tumours, i.e. those with extension of disease outside the tissue of origin, had a much poorer prognosis with an actuarial 5-year survival rate of only 21%. Two other factors, histological type and site of primary tumour, appeared to affect prognosis but were not independent of the extent of disease at diagnosis.

All children were treated according to protocol. Fifty-two patients showed a complete response to initial therapy and 4 of the 11 partial responders achieved a full remission after additional therapy. The overall complete response rate was therefore 77%. Nineteen children who achieved a complete response on initial treatment subsequently relapsed. Only 3 of these children were alive with no evidence of disease 3 years later, a salvage rate of 15%. "Late" relapses, defined as those occurring more than 2 years after diagnosis, were seen in only 5 children, 4 in boys with primary paratesticular tumours.

The protean manifestations of rhabdomyosarcoma in childhood render it a fascinating and challenging tumour to diagnose and treat. Rhabdomyosarcoma constitutes about 4% of all the malignant tumours seen in childhood and in England, Scotland and Wales, approximately 50 new cases occur annually in children under the age of 15 years (Draper *et al.*, 1982). The primary tumour develops in many sites but most frequently in the head and neck region, urogenital tract, trunk and extremities. Rhabdomyosarcoma is a highly malignant tumour with a tendency to infiltrate adjacent structures and to metastasise to lungs, bone, lymph nodes and sometimes to bone marrow.

During the 1960's treatment with surgery and radiotherapy, either alone or in combination, produced 5-year survival rates of 14–35% (Ehrlich *et al.*, 1971; Kilman *et al.*, 1973; Maurer *et al.*, 1977; Sutow *et al.*, 1970). The introduction of multiple agent chemotherapy into treatment protocols during the early 1970's resulted in a considerable improvement in prognosis. Using the multimodality approach to treatment of rhabdomyosarcoma, disease-free survival rates of 90% at 3 years have been reported for patients presenting with non-metastatic disease in sites such as the orbit and genito-urinary tract. (Hays *et al.*, 1981; Sutow *et al.*, 1982).

In January 1974 the Children's Solid Tumour Group (CSTG) was set up between St. Bartholomew's and the Royal Marsden Hospitals, with the objectives of improving the understanding of the biology of the solid tumours of childhood and of developing more effective treatment protocols.

### Patients and methods

This report describes the experience of the CSTG for the period February 1974 to December 1981, during which time 73 children with rhabdomyosarcoma were treated by members of the group. The analysis of survival and relapse rates includes events occurring up to the end of December 1982.

### *Pathology*

In all cases the diagnosis was confirmed by histological examination of the tumour tissue. Routine stains of the histological sections included haematoxylin and eosin, periodic acid schiff and

Correspondence: J.E. Kingston
*Members of the CSTG: A. Barrett[1], H.J.G. Bloom[1], J. Freeman[4], J. Graham-Pole[3], J.M. Henk[1], D.N. Lawson[1], T.J. McElwain[1], J.S. Malpas[2] & M.R. Sandland[5].
[3]Department of Pediatrics, University of Florida, Gainesville, Florida 32610. [4]Cancer Therapy and Research Foundation of South Texas, U.S.A. and [5]Peter McCallum Institute, Melbourne, Victoria, Australia.

Received 25 February 1983; accepted 6 May 1983.

© The Macmillan Press Ltd., 1983

phosphotungstic acid haematoxylin. Since 1977, tumour tissue has been examined by electron microscopy whenever possible. Fifty nine patients had their initial biopsy or surgery carried out at the referring hospital and the pathological material of all these cases was reviewed by a senior consultant pathologist at one of the two collaborating hospitals. Nineteen (32%) of these 59 cases had been referred with a different diagnosis, which was revised to rhabdomyosarcoma following review of the histology. This finding emphasises the need for these relatively uncommon tumours to be examined by a pathologist with considerable experience in the field of paediatric oncology.

The histological subtypes included in this study are those classified as embryonal sarcoma and the embryonal, alveolar and botryoid types of rhabdomyosarcoma. Two cases where the type of rhabdomyosarcoma could not be determined are also included. No cases of pleomorphic rhabdomyosarcoma have been seen.

*Staging*

One of the objectives of the study was to relate the extent of the disease at diagnosis to outcome. Three different staging systems were used in an attempt to determine a classification which would give a good differential with regard to outcome. The three staging systems which have been used are as follows:

1. St. Jude (Pratt *et al.*, 1972)
2. T.N.M. (UICC, 1982)
3. Barts/Marsden

All 3 systems refer to the pre-treatment clinical assessment of the disease state and are outlined in Tables I–III.

*Staging investigations*

After histological confirmation of the diagnosis, the investigations done as part of the initial staging assessment on all patients included full blood count, chest X-ray, bone scan, bone marrow aspirate and trephine. Other investigations such as abdominal ultrasound, C.T. scanning, i.v. urography, lymphangiography and lumbar puncture were done when appropriate.

*Definition and evaluation of response*

(1) Complete response (CR):
disappearance of all evidence of disease for more than one month

(2) Good partial response (GPR):
>50% reduction in size of tumour at the primary site and clearance of metastatic disease for more than one month

(3) Poor partial response (PPR):
<50% reduction in size of tumour at the primary site but clearance of all metastatic disease for more than one month

(4) No response (NR):
persistence or progression of disease.

**Table I** St. Jude staging system

| *Stage* | *Description* |
|---|---|
| I | Localised disease —completely resectable |
| II | Regional disease |
| A | —completely resectable |
| B | —nonresectable or partially resectable |
| III | Generalised disease |
| A | —distant metastases with normal bone marrow |
| B | —distant metastases with positive bone marrow |

**Table II** TNM Staging system

| *T* *Primary tumour* | *N* *Regional lymph nodes* | *M* *Distant metastases* |
|---|---|---|
| $T_1$ Tumour confined to the organ or tissue of origin | $N_0$ No evidence of regional lymph node involvement | $M_0$ No evidence of distant metastases |
| $T_{1a}$ Tumour 5 cm. or less | $N_1$ evidence of regional lymph node involvement | $M_1$ evidence of distant metastases |
| $T_{1b}$ Tumour more than 5 cm | | |
| $T_2$ Tumour involving contiguous organs or tissues or with adjacent malignant effusion. | | |

**Table III** Barts/Marsden staging system

| Stage | Description |
|---|---|
| I | Localised disease confined to tissue of origin |
| | A —completely resected |
| | B —not resected |
| II | Regional disease |
| | A —extending outside tissue of origin to involve contiguous bone or nerve |
| | B —with nodal metastases |
| III | Generalised disease |
| | A —without marrow involvement |
| | B —with marrow involvement |

The nature of the investigation chosen to evaluate response depended on the site and stage of the disease. Methods of evaluation have included clinical examination, ultrasound and C.T. scanning, bone marrow trephine, lumbar puncture and repeat biopsy at the primary site. Reappearance or progression of the disease after a partial or complete response has been defined as a relapse.

*Treatment protocols*

Between February 1974 and January 1977 the allocation of patients to a treatment protocol was determined by the stage of the disease, based on the St. Jude staging system. All children received intensive chemotherapy with vincristine, actinomycin-D and cyclophosphamide for one year and in addition children with non-metastatic disease were given radical radiotherapy, (40–60 Gy) to the site of the primary tumour. This treatment regimen is outlined in Table IV.

From February 1977 onwards, treatment was intensified by the addition of adriamycin for "poor risk" cases, i.e. children with Stage IIB tumours in parameningeal sites and all Stage III cases. Children at the Royal Marsden Hospital with rhabdomyosarcoma of the orbit were also treated with adriamycin but on a slightly modified regimen. These revised protocols are outlined in Table V.

*Statistical analysis*

Standard Kaplan Meier methods have been used for calculating and projecting curves of overall survival and periods of disease-free survival. Tests of statistical differences between curves have been analysed using a standard log rank test.

### Results

A total of 73 children with rhabdomyosarcoma and embryonal sarcoma have been treated during the period February 1974–December 1981. Early results of treatment of 11 of these children have been reported previously (Malpas *et al.*, 1976).

*Primary site*

The distribution of tumours by primary site is shown in Table VI. The commonest site of disease in our series was the orbit, accounting for 21% of the total. The relatively high number of children with orbital lesions may reflect local referral patterns resulting from the close association of the two collaborating hospitals with the country's major specialist eye hospital. It is estimated that 40–50% of all cases of orbital rhabdomyosarcoma in England and Wales are treated by members of the CSTG.

**Table IV** Treatment protocol (Feb. 1974–Jan. 1977)

| Stage (St. Jude) | Surgery | Radiotherapy | Chemotherapy |
|---|---|---|---|
| I | Complete excision with resection of | 4000–6000 cGy over 6–7 weeks | VAC* |
| II A | adjacent lymph nodes in Stage IIA | | |
| II B | Partial excision or biopsy with determination of extent of tumour | 4000–6000 cGy over 6–7 weeks | VAC |
| III A | Biopsy only | Palliative RT to bulky disease | VAC |
| B | | | |

*VAC—Vincristine 1.5 mg m$^{-2}$ i.v.
Actinomycin D 0.6 mg m$^{-2}$ i.v.
Cyclophosphamide 300 mg m$^{-2}$ i.v.
Courses of chemotherapy given weekly ×6 concurrently with radiotherapy and then fortnightly for one year.

**Table V** Treatment protocol (Feb. 1977–Dec. 1981)

| *Stage (St. Jude)* | *Surgery* | *Radiotherapy* | *Chemotherapy* |
|---|---|---|---|
| Good risk | | | |
| I<br>IIA<br>IIB—other than para-meningeal sites | Complete excision with resection of adjacent lymph nodes in Stage IIA<br>Biopsy only in Stage IIB | 4000–6000 cGy to site of primary tumour | (a) 2 courses of VAC* prior to RT<br>(b) starting 2 weeks after RT-VAC at 3 week intervals to 1 year |
| Orbital tumours (RMH) | Biopsy | ,, | (a) 2 courses of VAC prior to RT<br>(b) VC† during RT<br>(c) VAC, VAC, VA¶ given at 3 week intervals for 9 cycles post RT |
| Poor risk | | | |
| IIB—para-meningeal sites | Biopsy | 4000–6000 cGy to residual tumour after chemotherapy and to sites where tumour existed prior to chemo-therapy | (a) 2 courses of CVA** prior to RT<br>(b) VC during RT<br>(c) starting 2 weeks after RT CVA 2 weekly ×8 followed by VAC at 2 week intervals for 1 year. |
| III A+B | ,, | | |

*VAC See **Table IV**.

| | | |
|---|---|---|
| **CVA | Vincristine | $1.5\,mg\,m^{-2}$ i.v. |
| | Cyclophosphamide | $400\,mg\,m^{-2}$ i.v. |
| | Adriamycin | $40\,mg\,m^{-2}$ i.v. |
| †VC | Vincristine | $1.5\,mg\,m^{-2}$ i.v. |
| | Cyclophosphamide | $200\,mg\,m^{-2}$ i.v. |
| ¶VA | Vincristine | $1.5\,mg\,m^{-2}$ i.v. |
| | Adriamycin | $40\,mg\,m^{-2}$ i.v. |

The relatively small number of very young boys with pelvic primary tumours included in this study is because these patients are usually referred to the Consultant Paediatric Urologist at the Hospital for Sick Children, Great Ormond Street.

### *Histological type*

The distribution of cases by histological type is shown in Table VII. The embryonal/botryoid classification accounted for 76% of the cases. The alveolar type (22%) was seen predominantly in limb, pelvic and perineal sites. This predominance of alveolar histology in extremity lesions was also reported by the Intergroup rhabdomyosarcoma study IRS-1, (Hays *et al.*, 1982*a*).

### *Age distribution*

The frequency of rhabdomyosarcoma by age is shown in Figure 1. Seventy three per cent of patients were aged <10 years at diagnosis. The median age was 6 years 2 months with a range of 2 weeks to 15 years 11 months.

### *Stage*

Classification of patients by the three staging systems is shown in Table VIII. Twenty children (28%) had tumours which were confined to the tissue of origin and completely resected (Barts/Marsden (BM) Stage IA), while a further 20 children had locally confined disease which was

**Table VI** Distribution of patients by site

| *Site* | | *No. of patients* |
|---|---|---|
| Orbit | | 16 |
| Other head and neck | | 9 |
| Parameningeal sites | | |
| —nasopharynx | (3) | |
| —para nasal sinus | (1) | |
| Face | (3) | |
| Neck/Larynx | (2) | |
| Paratesticular | | 11 |
| Vagina | | 6 |
| Other pelvic/retroperitoneal sites | | 15 |
| Pelvis | (9) | |
| Perineal | (3) | |
| Prostate | (1) | |
| Retroperitoneum | (2) | |
| Extremity | | 4 |
| Lower limb | (3) | |
| Upper limb | (1) | |
| Trunk/Limb girdle | | 5 |
| Intrathoracic | | 3 |
| C.N.S. | | 2 |
| Primary site not identified | | 2 |
| | Total | 73 |

**Figure 1** Distribution of patients by age at diagnosis. Shaded areas represent children with alveolar histology.

**Table VII** Distribution of patients by histological type

| *Tumour histology* | *No. of patients* | *% of total* |
|---|---|---|
| Embryonal/botryoid | 38 | 52 |
| Embryonal sarcoma | 17 | 24 |
| Alveolar | 16 | 22 |
| Rhabdomyosarcoma (n.o.s.) | 2 | 2 |

**Table VIII** Distribution of patients by stage

| *Barts/Marsden* | | *TNM* | | *St. Jude* | |
|---|---|---|---|---|---|
| *Stage* | *No. patients* | *Stage* | *No. patients* | *Stage* | *No. patients* |
| IA | 20 | $T_{1a}N_0M_0$ | 28 | I | 17 |
| IB | 20 | $T_{1a}N_1M_0$ | 3 | IIA | 3 |
| IIA | 9 | $T_{1b}N_0M_0$ | 11 | IIB | 39 |
| IIB | 10 | $T_{1b}N_1M_0$ | 4 | IIIA | 7 |
| IIIA | 7 | $T_{1b}N_0M_1$ | 5 | IIIB | 7 |
| IIIB | 7 | $T_{1b}N_1M_1$ | 4 | | |
| | | $T_2N_0M_0$ | 9 | | |
| | | $T_2N_1M_0$ | 4 | | |
| | | $T_2N_0M_1$ | 5 | | |

either partially resected or merely biopsied (BM Stage IB). In 9 children without nodal or metastatic disease, the tumour extended outside the tissue of origin to involve contiguous bone (BM Stage IIA). Ten children had lymph node involvement but no other evidence of systemic spread (BM Stage IIB). Four of these children however had evidence of local infiltration. The remaining 14 children had metastatic disease which, in 7, involved the bone marrow.

When patients are staged according to the St. Jude system, there is a rather uneven distribution of cases between the five stages, with a large proportion (52%) falling into Stage IIB.

Lymphatic spread was not seen in any of the 16 children with orbital tumours, but was common in other head and neck sites (4/9). No lymphatic involvement was observed in any of the children with lesions of the trunk. The relative infrequency of lymphatic spread in patients with orbital and truncal lesions has also been noted by the Inter Group Rhabdomyosarcoma Study (Donaldson *et al.*, 1973; Lawrence *et al.*, 1977).

### *Survival analysis*

The median duration of follow up for this group of patients is 49 months with a range of 14–103 months. The predicted actuarial 5-year survival rate for all patients is 58% with a disease-free survival rate of 45% (Figure 2). No relapse or death has been observed more than 4 years after diagnosis.

**Figure 2** Actuarial survival curves for children with rhabdomyosarcoma treated at St. Bartholomew's and The Royal Marsden Hospitals between January 1974 and December 1981.

*Effect of stage*

The survival curves for patients staged according to the St. Jude system are shown in Figure 3. For Stage I patients the actuarial 5-year survival rate is 73%, compared to 65% for all Stage II patients. The difference between these survival rates is not significant ($P = 0.39$). It would therefore appear that the St. Jude staging system does not give a good differential with regard to outcome between Stage I and II patients.

Survival curves for patients staged according to the TNM system are shown in Figure 4. These show that the patients with the best prognosis are those with tumours classified as $T_1$ $N_0$ $M_0$ for whom the actuarial survival rate at 5 years is 85%. However patients with $T_2$ $N_0$ $M_0$ tumours, i.e. those with tumours extending outside the tissue of origin, but without nodal or metastatic disease, fared badly, with a survival rate of only 25%. Similarly, patients with nodal involvement but no evidence of metastatic spread, $T_{1+2}$ $N_1$ $M_0$ did badly with a predicted 5-year survival rate of 30%. However, excluding the children with $T_2$ tumours in this group, patients with nodal spread but locally confined tumours $T_1$ $N_1$ $M_0$ had an intermediate prognosis with a 48% survival rate at 5 years.

Survival curves for patients staged according to the Barts/Marsden system are shown in Figure 5. There is a predicted actuarial 5-year survival rate of 86% for all Stage I patients, 78% and 95% for Stage IA and IB respectively. This difference is not significant ($P = 0.25$) It therefore appears that surgical resection of locally-confined tumours does not make a significant difference to overall survival. This finding is of particular relevance for patients with orbital rhabdomyosarcoma for whom total excision of the tumour with enucleation used to be the treatment of choice. The poor survival rate of the Barts/Marsden Stage II patients (26%), with rates of 22% and 40% for BM Stages IIA and IIB respectively, means that both local extension of the tumour outside the tissue of origin and nodal spread are bad prognostic features.

In all three staging systems patients with metastatic disease do badly, with a survival rate of $<15\%$ at 2 years. Of the 14 patients with generalised disease at presentation only one child is alive and disease free at 3 years from diagnosis.

*Effect of site*

The best survival rates were seen in children with tumours of the orbit, the majority of whom had disease confined within the orbit. These children had a predicted 5-year survival rate of 94%. Children with tumours in other head and neck sites did less well with a survival rate of 50%. Boys with paratesticular tumours and girls with vaginal tumours also tended to have a good prognosis with 5-year survival rates of 81% and 67% respectively. Children with tumours in other pelvic sites did

**Figure 3** Overall survival of children with rhabdomyosarcoma staged according to the St. Jude staging system.

**Figure 4** Overall survival of children with rhabdomyosarcoma staged by the T.N.M. classification.

**Figure 5** Overall survival of children with rhabdomyosarcoma staged according to the Barts/Marsden staging system.

**Figure 6** Effect of site of tumour on overall survival in childhood rhabdomyosarcoma.

particularly badly, with a predicted survival rate of only 31% at 5 years. Children with limb and trunk primaries had an intermediate prognosis, 44% at 5 years (Figure 6).

*Effect of age*

Age did not appear to have any significant effect on prognosis, and we did not find that infants have a worse prognosis than older children (Grosfield *et al.*, 1969). Of the 5 children diagnosed under the age of one year, all are currently alive and disease-free, with a median follow up of 77 months (range 57–91). Three of these 5 infants were girls with vaginal botryoid tumours.

*Effect of sex*

Forty eight per cent of the female children have died compared to only 23% of the male patients, with survival rates of 69% and 44% for males and females respectively. The poorer survival rates for females may, in part, be explained by the higher incidence in this series of girls with pelvic tumours and with tumours in head and neck sites. The difference in survival between the sexes just fails to reach statistical significance ($P = 0.067$), (Figure 7).

*Effect of histology*

Children with tumours classified as embryonal rhabdomyosarcoma or embryonal sarcoma had a better prognosis than those with tumours of alveolar histology; 66% survival at 5 years for those with embryonal histologies, compared to only 25% for those with alveolar histologies ($P < 0.001$), (Figure 8).

*Response to treatment*

Eleven children required significant modifications to planned treatment because of drug toxicity or profound bone marrow suppression. Exclusion of a drug, reduction of drug dose by more than 33% of the planned dose, or a delay of more than 3 weeks in administering the drug were all considered as significant modifications to protocol. Of the 73 children, 52 (71%) achieved a complete remission on initial therapy, 6 patients achieved a good partial remission while a further five showed only a poor partial response.

Four of the partial responders achieved a complete remission on subsequent therapy, and of these, 3 are alive and disease-free at intervals ranging from 13 months to 6 years. The overall complete response rate was therefore 77%. No child who failed to achieve a complete response is a long term survivor.

Ten patients failed to respond to initial therapy and of these, 6 had metastatic disease at diagnosis and the other 4 had evidence of nodal spread and/or locally invasive disease. The median duration of survival for the non responders was 24 weeks. Only 4 of the 14 children with metastatic disease at diagnosis achieved a complete remission and of

**Figure 7** Effect of sex on overall survival in childhood rhabdomyosarcoma.

**Figure 8** Effect of histology on overall survival in childhood rhabdomyosarcoma.

these only one patient, a boy with a paratesticular primary who had bone marrow involvement at presentation, is currently alive with no evidence of disease at 3 years from diagnosis.

*Relapses*

Although 52/73 patients achieved a complete remission on initial therapy, 19 (37%) of these complete responders have subsequently relapsed and the relapse free survival is 45%. The median time to first relapse for children achieving complete remission on initial therapy was 14.5 months, which is similar to the findings of other series (Heyn *et al.*, 1974). At relapse, 8 of the 19 children had disease confined to the primary site, 8 developed distant metastases with no sign of recurrent disease at the primary site while 3 children had evidence both of metastatic disease and of local recurrence. Ten of these 19 relapses occurred in girls. Taking into account the smaller number of girls in the series (33 girls *vs* 40 boys) there was a slight excess of girls amongst relapsing patients. This has also been observed by Niefeld *et al.* (1979).

The median duration of survival from the time of first relapse was 6 months, with a range of 2 weeks to 70 months. Nine (47%) of the 19 children who relapsed, achieved a second complete remission with further therapy, whilst a further 2 patients are currently on treatment following a recent relapse. Four of the 9 children who achieved a complete response following a first relapse have had a second relapse, after disease free intervals ranging from 4 to 16 months. The other 5 children remain well and disease free at intervals ranging from 6 months to nearly 6 years. Five children have relapsed after more than 2 years following diagnosis, and of these, 4 have been boys with paratesticular primaries. The latest time at which a first relapse has been observed, occurred at 3 years from diagnosis.

### Discussion

Radical changes in the management of rhabdomyosarcoma have occurred over the past decade and greater reliance is now placed on radiotherapy and chemotherapy to control local disease in sites where previously radical surgery would have been the treatment of choice. It is generally accepted that a multi-modality approach to the management of rhabdomyosarcoma provides the best chance of long term survival. However, the optimum duration and combination of therapies remain subjects of considerable debate.

The interplay of a variety of factors including stage, size, site, sex and histological type makes it extremely difficult to study the effect of different therapeutic protocols on outcome. However, there is little doubt that the extent of disease at diagnosis exerts the greatest influence on prognosis. Unfortunately, any comparison of survival rates between series from different institutions has been confounded by the fact that there is no universally accepted staging system for rhabdomyosarcoma. Due to the changing emphasis in treatment, the St. Jude system, first described by Pratt (1969) now seems inappropriate for many primary sites.

The CSTG has defined a clinical classification, the Barts/Marsden staging system, which is similar to that described by Jaffe *et al.* (1973). This system incorporates some of the more important concepts of both the St. Jude and the TNM classifications and appears to give a good differential with regard to outcome. In this series it has clearly shown that the children with the best prognosis are those with locally "confined" tumours (BM stages IA and IB) i.e. tumours which have not spread to nodes or distant sites and which have not infiltrated contiguous tissues. It has also shown that complete surgical resection of locally "confined" tumours does not improve prognosis as we have found no significant difference in outcome between patients with Stage IA and IB tumours. The Barts/Marsden system defines a subgroup of children who have tumours extending outside the tissue of origin to infiltrate contiguous tissue, usually bone (BM Stage IIA). These children do badly on standard treatment and should therefore be classified as "poor risk" patients. The system also shows that children with nodal involvement (BM Stage IIB) fare badly and confirms the findings of other classification systems that children with metastatic disease at diagnosis (BM Stage IIIA and IIIB) have a very poor prognosis.

The overall actuarial 5-year survival rate for children in our series was 58%, ranging from <15% in children with metastatic disease to 86% in children with locally confined tumours. These results are similar to those reported by the Intergroup Rhabdomyosarcoma Study Group (Maurer *et al.*, 1977).

In our series, the orbit was the most common primary site, and the children with tumours in this site were found to have the best prognosis, with a survival rate of 94% at 5 years. All but 2 of these children had Stage IA or B disease. This may reflect early detection of disease within the orbit. The paucity of lymphatics within the orbit may explain the infrequency of lymphatic spread.

During the 1960's a radical surgical approach with exenteration was recommended for rhabdomyosarcoma of the orbit. This present series confirms the finding of Donaldson *et al.* (1973) that biopsy followed by intensive chemotherapy and irradiation can result in long term disease control, thereby avoiding the necessity for exenteration, a severely mutilating operation. Only one child has developed serious ocular complications as a result of radiation treatment; in this case enucleation was unfortunately required because of severe keratitis. One other child has required a subsequent exenteration because of recurrent disease at the primary site.

Rhabdomyosarcoma of the urogenital tract was the second commonest primary site in this series. Boys with paratesticular tumours and girls with vaginal primaries both appeared to have a good prognosis, whereas children with perineal, prostatic and unspecified pelvic primaries fared much less well. Nodal involvement was seen in 24% of children with tumours of the urogenital tract and lymphangiography should therefore be considered in all patients with tumours originating in these sites. In 1977 the committee for the Intergroup Rhabdomyosarcoma Study reported a 19% incidence of regional node involvement in children with genito-urinary rhabdomyosarcoma (Lawrence *et al.*, 1977) and a subsequent report from the group (Tefft *et al.*, 1980) described positive node involvement in 15/38 children who had undergone regional node sampling.

It is of interest that there appears to be a trend for girls to do less well than boys, although in this series the difference in survival fails to reach statistical significance. However, a similar trend which is statistically significant has been observed in an analysis of the national data collected by the Childhood Cancer Research Group in Oxford. (Stiller, 1982 personal communication). In our series, the poorer prognosis for females appeared to be related to the higher proportion of females with tumours in unfavourable sites i.e. pelvic and non-orbital head and neck sites, who had more extensive disease at diagnosis. However, those findings were not corroborated in the national series, where an even distribution between the sexes was found for these sites.

In our series, children with tumours of alveolar histology, had a much poorer prognosis than those with embryonal histology with survival rates of 68% and 25% for embryonal and alveolar histologies respectively. This finding is similar to that observed by the Intergroup rhabdomyosarcoma study who reported a mortality of 62% in patients with alveolar histology compared to 38% in children with embryonal histology (Hays *et al.*, 1982*b*). However, in our series the poor prognosis of children with tumours of alveolar histology was also related to the stage of the disease, as 87% of the children with alveolar histology had "unconfined" tumours (BM stages II and III) compared to only 33% of children with embryonal histology. It would therefore appear that tumours of alveolar histology have a tendency to infiltrate locally and to metastasise to distant sites.

Although 77% of the patients eventually achieved a complete response, it was disappointing to find that over a third (37%) of these patients subsequently relapsed. Recurrent disease at the primary site was present in 58% of the patients who relapsed. The long term salvage rate following a relapse was only 15%. The median time to first relapse was 14.5 months, and it is chastening to find that there are a significant number of relatively "late" relapses, 5 out of 19 occurring more than 2 years from diagnosis, emphasising the need for long term surveillance of these children (Maurer *et al.*, 1977).

The advent of CT scanning has made a considerable contribution to the management of rhabdomyosarcoma and embryonal sarcoma, both in the initial assessment at diagnosis and in subsequent evaluation and follow up. It has been of particular value in children with primary lesions in the pelvis and nasopharynx, where it has been able to demonstrate the very extensive nature of many of the tumours in these sites. A more aggressive approach in the treatment of tumours of the nasopharynx was introduced by the CSTG in 1977, but as yet the number of children treated on the more intensive protocol is too small to say whether the intensification of therapy has made a significant impact on prognosis. Similarly children with pelvic primaries require aggressive therapy to achieve control of their disease, and the results of our recent treatment protocols for tumours in these sites remain disappointingly poor.

In conclusion, it is encouraging that considerable improvements in prognosis have resulted from the introduction of a multimodality approach to the treatment of rhabdomyosarcoma and embryonal sarcoma. Eighty-five per cent of children with locally "confined" tumours can now expect to survive for 5 years and are hopefully cured of their disease. Unfortunately, the prognosis for children with "unconfined" disease remains extremely poor, and it is in these children that new therapeutic approaches are required. Responses to high dose chemotherapy have been seen in children with relapsed rhabdomyosarcoma resistant to conventional doses of cytotoxic agents. Members of the CSTG are therefore currently assessing the value of high dose melphalan with autologous marrow rescue, used in an adjuvant role following 6 courses of chemotherapy at conventional dosage, for patients defined as poor risk i.e. those with "unconfined" or advanced stage disease.

We thank Prof. R.P. Jackson and Mr. W. Gregory at the University of Warwick for statistical advice and help with the computing aspects of this study, Dr. A.G. Stansfeld for reviewing the histology of the cases treated at St. Bartholomew's Hospital and Mrs. P. Holloway in her capacity as Research secretary to the CSTG.

### References

DONALDSON, S.S., CASTRO, J.R., WILBUR, J.R. & JESSE, R.H. (1973). Rhabdomyosarcoma of head and neck in children—combination treatment by surgery, irradiation and chemotherapy. *Cancer*, **31**, 26.

DRAPER, G.J., BIRCH, J.M., BITHELL, J.F. & 6 others. (1982). Childhood cancer in Britain—incidence, survival and mortality. *Studies on Medical and Population Subjects*. No. **37**, H.M.S.O.

EHRLICH, F.E., HAAS, J.E. & KIESWETTER, W.B. (1971). Rhabdomyosarcoma in infants and children: factors affecting long-term survival. *J. Pediatr. Surg.*, **6**, 571.

GROSFIELD, J.L., CLATWORTHY, W. & NEWTON, W.A. (1969). Combined therapy in childhood rhabdomyosarcoma—an analysis of 42 cases. *J. Pediatr. Surg.*, **4**, 637.

HAYS, D.M., RANEY, R.B., LAWRENCE, W., MAURER, H.M. & TEFFT, M. (1981). Rhabdomyosarcoma of bladder and prostate in children. *J. Pediatr. Surg.*, **16**, 928.

HAYS, D.M., SOULE, E.H., LAWRENCE, W. & 5 others. (1982*a*). Extremity lesions in the intergroup rhabdomyosarcoma study (IRS-1). A preliminary report. *Cancer*, **48**, 1.

HAYS, D.M., NEWTON, W., SOULE, E.H. & 5 others. (1982*b*). Influence of alveolar histology on survival in the intergroup rhabdomyosarcoma study (IRS-1). *Proc. Am. Soc. Clin. Oncol.*, **1**, 176 (Abstract).

HEYN, R.M., HOLLAND, R., NEWTON, W.A., TEFFT, M., BRESLOW, N. & HARTMAN, J.R. (1974). The role of combined chemotherapy in the treatment of rhabdomyosarcoma in children. *Cancer*, **34**, 2128.

JAFFE, N., FILLER, R.M., FARBER, S. & 4 others. (1973). Rhabdomyosarcoma in children. Improved outlook with a multidisciplinary approach. *Am. J. Surg.*, **125**, 482.

KILMAN, J.W., CLATWORTHY, H.W., NEWTON, W.A. & GROSFIELD, J.L. (1973). Reasonable surgery for rhabdomyosarcoma. *Ann. Surg.*, **178**, 346.

LAWRENCE, W., HAYS, D.M. & MOON, T.E. (1977). Lymphatic metastasis with childhood rhabdomyosarcoma. *Cancer*, **39**, 556.

MALPAS, J.S., FREEMAN, J.E., PAXTON, A., WALKER-SMITH, J., STANSFELD, A.G. & WOOD, C.B.S. (1976). Radiotherapy and adjuvant combination chemotherapy for childhood rhabdomyosarcoma. *Br. Med. J.*, **i**, 247.

MAURER, H.M., MOON, T.E., DONALDSON, M. & 11 others. (1977). The intergroup rhabdomyosarcoma study. A preliminary report *Cancer*, **40**, 2015.

NIEFELD, J.P., MAURER, H.M., GODWIN, D., BERG, J.W. & SALZBERG, A.M. (1979). Prognostic variables in paediatric rhabdomyosarcoma before and after multimodal therapy. *J. Paediatr. Surg.*, **14**, 699.

PRATT, C.B. (1969). Response of childhood rhabdomyosarcoma to combination chemotherapy. *J. Paediatr.*, **74**, 791.

PRATT, C.B., HUSTU, H.O., FLEMING, I.D. & PINKEL, D. (1972). Co-ordinated treatment of childhood rhabdomyosarcoma with surgery, radiotherapy and combination chemotherapy. *Cancer Res.*, **32**, 606.

SUTOW, W.W., SULLIVAN, M.P., RIED, H.L., TAYLOR, H.G. & GRIFFITH, K.M. (1970). Prognosis in childhood rhabdomyosarcoma. *Cancer,* **23**, 1384.

SUTOW, W.W., LINDBERG, R.D., GEHAN, E.A. & 4 others. (1982). Three year relapse-free survival rates in childhood rhabdomyosarcoma of the head and neck. *Cancer,* **49**, 2217.

TEFFT, M., HAYS, D.M., RANEY, R.B. & 5 others. (1980). Radiation to regional nodes for rhabdomyosarcoma of the genitourinary tract in children: is it necessary? A report from the intergroup rhabdomyosarcoma study (IRS-1) *Cancer,* **45**, 3065.

UICC-UNION INTERNATIONALE CONTRE LE CANCER (Geneva) (1982). TNM Classification of Paediatric Tumours p. 23.